# Immersion in nature enhances neural indices of executive attention

**DOI:** 10.1038/s41598-024-52205-1

**Published:** 2024-01-22

**Authors:** Amy S. McDonnell, David L. Strayer

**Affiliations:** Department of Psychology, University of Utah, Salt Lake City, UT 84102 USA

**Keywords:** Psychology, Psychology and behaviour

## Abstract

There is conjecture that our modern urban environments place high demand on our attentional resources, which can become depleted over time and cause mental fatigue. Natural environments, on the other hand, are thought to provide relief from this demand and allow our resources to be replenished. While these claims have been assessed with self-report and behavioral measures, there is limited understanding of the neural mechanisms underlying these attentional benefits. The present randomized controlled trial fills this gap in the literature by using electroencephalography to explore three aspects of attention—alerting, orienting, and executive control—from a behavioral and neural perspective. Participants (*N* = 92) completed the Attention Network Task before and after either a 40-min walk in nature or a 40-min walk in a control, urban environment. Participants that walked in nature reported their walk to be more restorative than those that walked in the urban environment. Furthermore, the nature group showed an enhanced error-related negativity after their walk, an event-related brain component that indexes executive control capacity, whereas the urban group did not. These findings demonstrate that a 40-min nature walk enhances executive control at a neural level, providing a potential neural mechanism for attention restoration in nature.

## Introduction

Our world is rapidly urbanizing. As of 2010, more people live in cities than in rural areas. The United Nations projects that by 2050, 70% of the world’s population will reside in urban centers^1^. While urbanization provides exposure to other cultures and access to education and health care, some characteristics of these environments (e.g., pollution, artificial light, stress, and overstimulation) negatively impact our health and cognition^2^. From an epidemiological standpoint, urban living is associated with higher rates of mood disorders^3^, aggression^4^, schizophrenia^5^, depression^6^, anxiety^7^, and posttraumatic stress disorder^8^. Furthermore, the modern human operates at an unsustainable pace. We are sensorily and attentionally overstimulated on a day-to-day basis, forced to shift our attention from one thing to the next with little time for recovery. Our workplaces and home environments are riddled with technology, as are the vehicles that transport us between the two. Text message pings, news alerts, and social media updates are a significant part of every day. Excessive technology use has been acknowledged by the World Health Organization as an area of public health concern^9^, as it is associated with negative moods^10^, stress^11^, and depletion of memory capacity^12^. The growing prevalence of smartphones and wearable devices mean that we are connected to technology at every given moment, making it difficult to concentrate on a single task or engage in meaningful face-to-face interactions. Importantly, this persistent over-stimulation and task-switching that is characteristic of our modern environments can deplete our attention and lead to mental fatigue^13,14^.

Exposure to nature is thought to buffer against the attentional detriments associated with modern living. In line with the above, Stephen Kaplan's Attention Restoration Theory (ART) posits that urban environments bombard our senses, placing constant demand on our attentional systems by forcing us to select elements that are useful and ignore those that are not^15^. This requires effortful, directed attention that is limited in capacity^16^ and can be depleted over time^17^, leading to impairments in executive functioning and self-regulation^18^. Kaplan postulates that spending time in nature allows the brain to rest and replenish directed attention. He argues that specific characteristics unique to nature—such as clouds, trees, water, and vast landscapes—can be restorative by recruiting more effortless, “involuntary” attention^19^. In other words, nature allows the mechanisms supporting effortful, directed attention to rest and recover as we effortlessly attend to the environment around us.

While many activities can be considered restorative (e.g., meditation, sleep, vacation), Kaplan outlines four qualifications that must be met for an environment to be particularly so. First, the environment must be *compatible* with the goals of the individual experiencing it. For example, an individual that is afraid of the dark may not find restoration on a camping trip. Second, the environment must allow for the sense of *being away*, in terms of either a physical or mental removal from the situation that demands attention and creates stress. Third, the environment must have *extent*, or a scope that allows for prolonged exploration both physically and conceptually. Lastly, there must be some level of engagement, which Kaplan calls “*soft fascination*”, that stimulates the senses in a bottom-up, sensory-driven fashion. For example, characteristics of nature like slow-moving clouds, sunsets, and trickling creeks engage the senses effortlessly. These four qualifications for restoration are characteristics of natural environments, thus allowing nature to serve as a place to rest and replenish our attentional resources^15^.

A large body of literature has accrued over the last three decades testing the claims set forth by Attention Restoration Theory^20,21^, with some studies supporting behavioral improvements in attention-related tasks after exposure to nature and others failing^22^ to do so. One reason for such mixed evidence is that there is conceptual ambiguity regarding which *aspects* of attention are most sensitive to depletion and subsequent restoration. Attention is multifaceted and thus there is inconsistency in the way researchers in the literature operationalize it. Attention is thought to be comprised of three anatomical networks that are responsible for different functions—alerting, orienting, and executive control^23,24^. Each of these functions serves a unique role in the way an individual attends to their environment. The alerting system helps achieve and maintain an aroused state over time, allowing us to stay awake, alert, and ready to respond to incoming stimuli^25^. This implicates neural regions related to arousal such as the locus coeruleus, thalamus, and frontal and parietal cortices^26^. The orienting system relates to visuospatial attention and affords the ability to select and prioritize important sensory information from specific locations in the environment. Orienting attention is associated with frontal eye fields^27^ and parietal cortices (such as the superior parietal lobe) that make up the dorsal attention network^28^. Lastly, the executive control system is responsible for higher-level cognitive functions, such as working memory and cognitive control. It helps in switching between different tasks and managing competing demands for attention, playing a role in resolving conflict and overriding pre-potent responses^23^. Executive control implicates the prefrontal cortex, the anterior cingulate cortex, and their associated circuitry^29,30^. Many have argued that executive control is most reflective of the concept of directed attention described in Attention Restoration Theory^18,21^ because “directed attention” and “executive control” both refer to the ability to focus attention while ignoring distractions^31^.

The present study explores which, if any, aspects of attention—alerting, orienting, or executive control—are most influenced by immersion in nature within a single task called the Attention Network Task (ANT). The ANT is designed to assess each aspect of attention independently^32^. It is a combination of the Posner spatial cueing task^33^ and the Flanker interference paradigm^34^ such that target flankers are either temporally or spatially cued. This task has high utility in the literature because not only does it provide a performance indicator of executive control (which is thought to closely capture the idea of directed attention), but it also provides performance indicators of non-executive control (alerting and orienting). This can help elucidate whether the attentional effects of exposure to nature are specific to a particular aspect of attention or if they encompass all three dimensions in a domain-general manner.

This is not the first study to measure ANT performance before and after exposure to nature (see Table 1); however, it does seek to overcome several limitations of the existing literature. Prior studies exhibit wide variability in results due to differences in sample sizes (in which they range from 12 to 60 participants), populations measured (children versus adults), whether participants had a clinical diagnosis (e.g., attention-deficit/hyperactivity disorder), experimental designs (within- versus between-subjects), type of nature (simulated nature versus real nature) and other methodological decisions that may influence results (e.g., whether participants were experimentally depleted before the walk and whether participants walked alone, with a group, or with a researcher). Therefore, the present experiment seeks to overcome this variation with a substantially larger sample size (92 participants), in a highly-controlled, randomized trial with both within- and between-subject components, comprised of a healthy adult sample. Additionally, participants were experimentally depleted at the start of testing to conceptually recreate Kaplan’s idea of the depleted cognitive state from which individuals may seek restoration. Furthermore, participants walked in real nature, enhancing the ecological validity of a literature that often relies on simulated nature. Lastly, participants in this study walked alone to allow for maximum restoration potential, as walking with other participants or researchers may be distracting or stressful, thus interfering with full cognitive immersion in the environment.

**Table 1:** Summary of prior attention network task studies in the attention restoration theory literature.

Authors	*N*	Population measured	Design*	Type of nature	Dose (min)	Control group	Experimental depletion	Results	Effect size
Berman et al. (2008)^35^	12	University students (mean age = 24.25 years)	Within-subjects	Images	10	Urban images	No	Improvement in executive control metric	Not reported
Gamble et al. (2014)^36^	56	30 older adults (mean age = 69.10 years) and 26 university students (mean age = 20.54 years)	Between-subjects	Images	6	Urban images	No	Nature imagery improved executive control metric for both age groups	Cohen’s *d* = 0.76
Emfield and Neider (2014)^37^	202	University students (mean age = 19.80)	Between-subjects	Sounds and/or Images	7–10	Urban sounds and/or images; no sounds or images	No	No changes in any ANT metrics	n/a
Bratman et al. (2015)^38^	60	University students and community adults (mean age = 22.90 years)	Between-subjects	Walk	50	Urban walk	No	No changes in any ANT metrics	n/a
Stevenson et al. (2019)^39^	33	Children (mean age = 12.03 years)	Within-subjects	Group walk with other participants and research team	30	Urban walk	Yes	Faster RTs after nature walk, but no changes in any ANT metrics	Cohen’s *d* = − 0.38
Stevenson et al. (2021)^40^	24	Children (mean age = 10.5 years) diagnosed with ADHD	Within-subjects	Walk with researcher	30	Urban walk + Medication dose	Yes	No changes in any ANT metrics	n/a
Johnson et al. (2021)^41^	31	University students (mean age = 20)	Within-subjects	Images	6	Urban images	No	No changes in any ANT metrics	n/a

*Within-subjects designs refer to studies where each participant was exposed to all experimental conditions (e.g., nature walk and urban walk). Between-subjects designs refer to studies where different participants were exposed to different experimental conditions (e.g., nature walk or urban walk).

Most importantly, the present study enhances existing work by co-registering behavioral and neurophysiological metrics generated from the ANT to assess all three aspects of attention from both a behavioral and neural level. Electroencephalography (EEG) is a neurophysiological method that records the electrical activity of the brain from electrodes placed on the scalp. It is non-invasive and allows for temporally precise, direct measurement of neural activity (on the order of milliseconds) in response to the demands of the environment. While some initial theorists proposed using EEG to explore nature’s influence on the brain^42^, there is still a relatively small literature that utilizes EEG compared to self-report, behavioral, and other neuroimaging methods such as functional magnetic resonance imaging^43,44^. Of the many ways to extract meaningful information from the EEG signal, researchers in this field quantify either continuous oscillatory activity in the frequency domain (e.g., alpha band activity from 8 to 12 Hz) or changes in electrical activity in response to discrete events in the environment in the time domain (i.e., event-related potentials).

Event-related potentials (ERPs) are valanced deflections in the EEG waveform that peak following the presentation of a stimulus (stimulus-locked ERPs) or following an individual’s response to a stimulus (response-locked ERPs). Importantly, they provide a direct, millisecond-resolution measure of brain activity and can provide insight into the neurophysiological correlates of both sensory and cognitive processes^45^. Compared to measuring power in certain frequency bands, ERP components have the advantage of high temporal precision and accuracy because they require minimal data processing or temporal filters^46^. Additionally, there is extensive literature surrounding the very specific antecedent conditions that elicit certain ERP components, making the interpretation of results relatively straightforward. The present study harnesses the high temporal precision of EEG and the strengths of the ERP technique to quantify neural metrics of alerting, orienting, and executive control generated from the Attention Network Task before and after exposure to nature compared to a control, urban environment. Co-registration of behavioral and neural metrics allows not only for information regarding task performance, but also the potential to identify neural mechanisms underlying changes in performance—insight that is largely absent from the Attention Restoration Theory literature.

Ninety-two adults took part in the study. Each participant was experimentally depleted at the start of testing with a counting backwards depletion task^14,47^. The intention behind a depletion manipulation was three-fold: (1) to conceptually recreate Kaplan’s idea of the depleted cognitive state from which individuals may seek restoration, (2) to prime participants for maximal restoration potential, and (3) to ensure that all participants entered their walk in a comparably depleted state. After cognitive depletion, participants completed the ANT while their behavioral performance and EEG signal were recorded. Participants were then randomly assigned to a 40-min walk in either a natural environment (46 participants) or an urban environment (46 participants) of comparable distance, elevation change, and environmental characteristics such as temperature, humidity, and wind speed. After the walk, each participant completed the ANT again, followed by a self-report measure of how restorative their walk was. We hypothesized that immersion in nature would be perceived as more restorative and would improve executive control metrics on the ANT, given executive control is thought to align closest with the construct of directed attention described in Attention Restoration Theory. We predicted that there would be no changes in alerting or orienting metrics.

## Methods and materials

This research complied with the American Psychological Association Code of Ethics and was approved by the Institutional Review Board at the University of Utah (IRB_00153144). All methods and procedures were performed in accordance with relevant guidelines and regulations of this institution. Informed consent was obtained from each participant.

### Participants

Participants (*N* = 92; 71 female, 20 male, 1 non-binary) between the ages of 18 and 57 (*M* = 29.43, *Mdn* = 26, *SD* = 10.52) were recruited via flyers, word of mouth, and the university participant pool. University-affiliated participants were an undergraduate sample of convenience, so participants from the community were also recruited to increase the generalizability of the results. 80% of participants identified as White, 10% as Asian, 7% as Latinx/Hispanic, and 3% as Black. Community participants were paid $50 USD for participating in the study and undergraduate students from the participant pool were granted 3 research credits for their participation.

Sample size was determined by an a priori power analysis conducted in PANGEA v0.2^48^, which indicated that 43 participants in each environmental condition (86 participants total) would provide sufficient power at the recommended 0.80 level to detect within-subject by between-group interactions based on a medium effect size (Cohen’s *d* = 0.50)^49,50^. This effect size was informed by prior research in the field that found medium-sized effects of nature exposure on indices of attention^36,51^. Data were collected from 92 participants to ensure that the sample size would not drop below the required 86 participants after anticipated data loss.

### Design

This randomized controlled trial employed a mixed design with both a within-subjects factor and a between-subjects factor. All participants completed the Attention Network Task before and after a 40-min walk; this repeated measure served as the within-subjects factor. Participants were randomized to walk in either a natural or an urban environment, serving as the between-subjects factor, while making sure that each gender was equally represented in each group.

### Procedure

**Figure 1:**
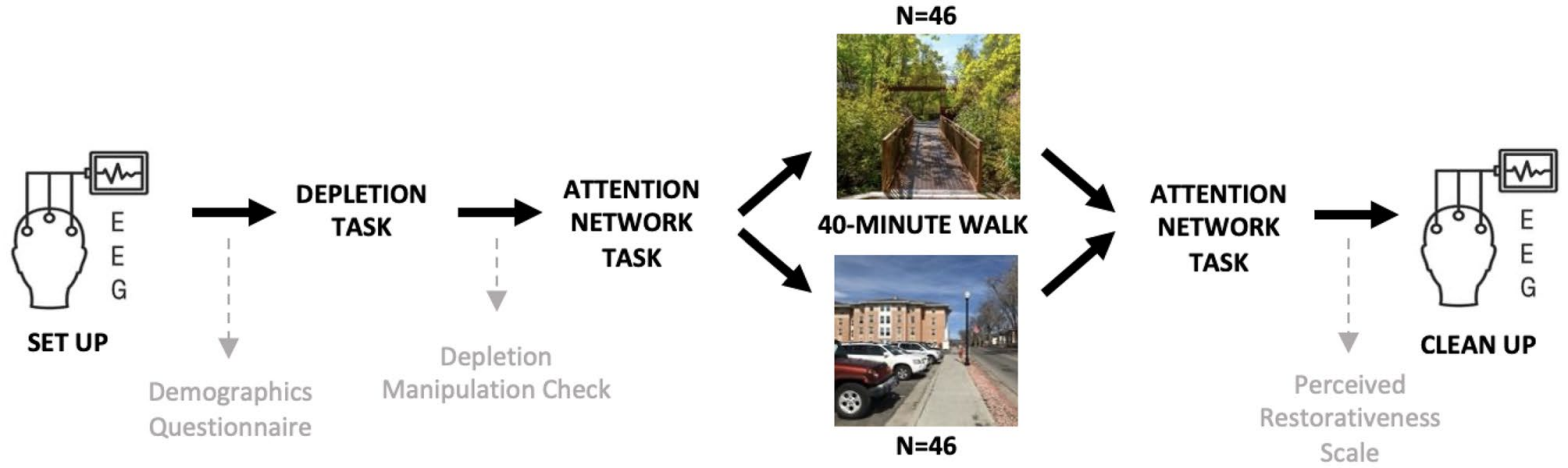
Procedural steps of the study. Self-report measures are written in grey. EEG was only recorded during the Attention Network Task.

**Figure 2:**
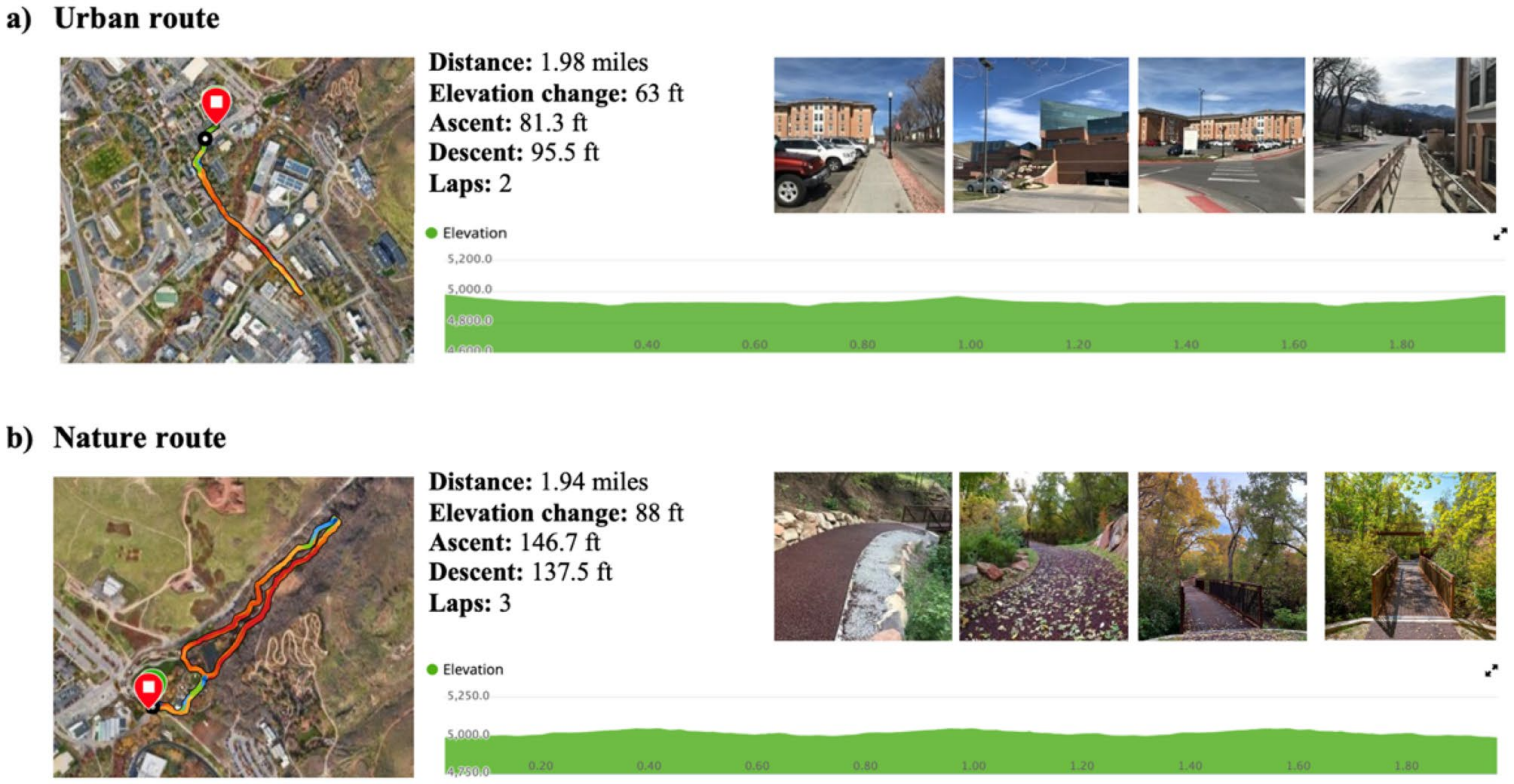
Maps, distances, elevation gradients, and photos of the two walking routes.

**Figure 3:**
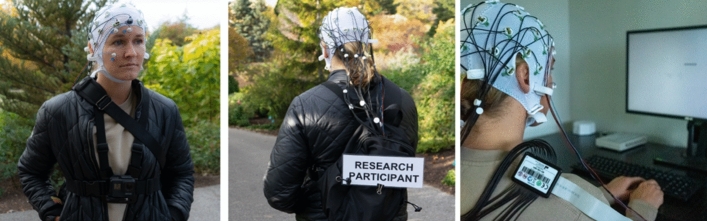
Left and middle: participant walking setup. See Supplemental Materials for GoPro camera data. Right: attention network task and EEG recording setup in the laboratory. Informed consent was obtained from the pictured participant for these images to be included.

All data collection and EEG recordings took place indoors in the Kay and Zeke Dumke, Jr. Horticulture Building at Red Butte Garden at the University of Utah, and both the nature and the urban walking routes left from this location. The procedural steps of the study can be visualized in Fig. 1. Upon arrival to the lab, participants completed a demographics survey while the researcher set up the EEG cap and electrodes. Once setup was complete, participants completed 10-min of a counting backwards task meant to deplete their cognitive resources. After depletion, participants completed the ANT for the first time. Participants were then randomly assigned to either a 40-min nature walk in an arboretum or a 40-min urban walk on an adjacent medical campus (see Fig. 2 for route information, Fig. 3 for participant walking setup, and Table 2 for additional information about the two groups). After the walk, participants returned to the lab and completed the ANT again, followed by the Perceived Restorativeness Scale to rate how restorative their walk was from a self-report standpoint. On average, the research procedure took 3 h to complete.

**Table 2:** Descriptive statistics of metrics generated from the Garmin GPS watch and environmental characteristics as a function of walk condition.

	Urban condition		Nature condition	
	Mean	SD	Mean	SD
Distance (miles)	1.99	0.04	1.93	0.13
Moving Time (min:s)	39:10	02:18	42:19	03:59
Pace (min:s/mile)	20:00	01:12	23:25	02:11
Avg. heart rate	110.41	15.03	108.24	16.13
Max. heart rate	137.26	18.44	131.71	17.42
Calories burned	199.85	40.74	217.27	45.27
Total ascent (ft)	123.37	76.69	146.00	49.89
Total descent (ft)	132.52	59.32	138.62	52.58
Temperature (°F)	72.28	12.59	74.36	12.88
Humidity (%)	30.52	11.68	29.84	12.40
Wind speed (mph)	8.20	4.27	8.31	5.39

### Walking routes and environmental characteristics

In both walk conditions, participants followed predetermined routes mapped by the research team. The two walking routes were of comparable distance (~ 2 miles) and elevation change (~ 60 to 90 feet). Participants were instructed to leave their cellphones and cellular data-connected watches in the research lab to prevent technological distraction and encourage engagement with their environment. While each participant was on their walk, the researcher recorded the current environmental characteristics (i.e., temperature, weather report, humidity, wind speed) to track some of the uncontrollable variables that may influence the walking experience. To control for any potential differences due to time of testing, nature and urban participants were equally distributed between morning and afternoon testing sessions such that 33 participants in each group testing in the morning and 13 participants in each group tested in the afternoon. For descriptive statistics of environmental conditions and metrics generated from the GPS watch as a function of walk condition, see Table 2.

In the nature walk condition, participants walked three laps around the route, and in the urban condition, participants walked two laps along the route. The “loop” approach was taken to reduce the cognitive load that may be attributed to navigating in a new environment, with the hope that after the first lap of route finding, participants would be able to enjoy their environment for the rest of the walk and not worry about navigating. The research team confirmed that each participant walked the correct route via the Garmin watch GPS data and GoPro video data.

### Depletion task

A counting backwards task known to induce high cognitive load was used to experimentally deplete participants and thus prime them for restoration^14,47^. For this task, participants were instructed to count backward from 1000 to 0 by 7’s for a total of ten minutes, and to do so out loud so that the researcher could ensure they were doing the task properly.

### Depletion manipulation check

Four manipulation check questions related to fatigue, effort, pleasantness, and frustration were administered immediately following the depletion task^52^. These manipulation check questions ensured that participants felt depleted after counting backwards and that reported levels of depletion were comparable across groups. See Supplemental Materials for the full set of questions.

### Perceived Restorativeness Scale

The 11-item version of the Perceived Restorativeness Scale (PRS-11) was administered at the end of the experimental protocol^53^. This scale assessed how restorative the participant perceived the environment they walked in to be. It includes eleven, 11-point Likert-scale ratings of statements such as “to stop thinking about the things that I must get done I like to go to places like this” and “to get away from things that usually demand my attention I like to go to places like this”. The scale is scored by calculating the average of the eleven items for each participant. A higher score represents greater perceived restoration. See Supplemental Materials for the full scale.

### Attention network task

**Figure 4:**
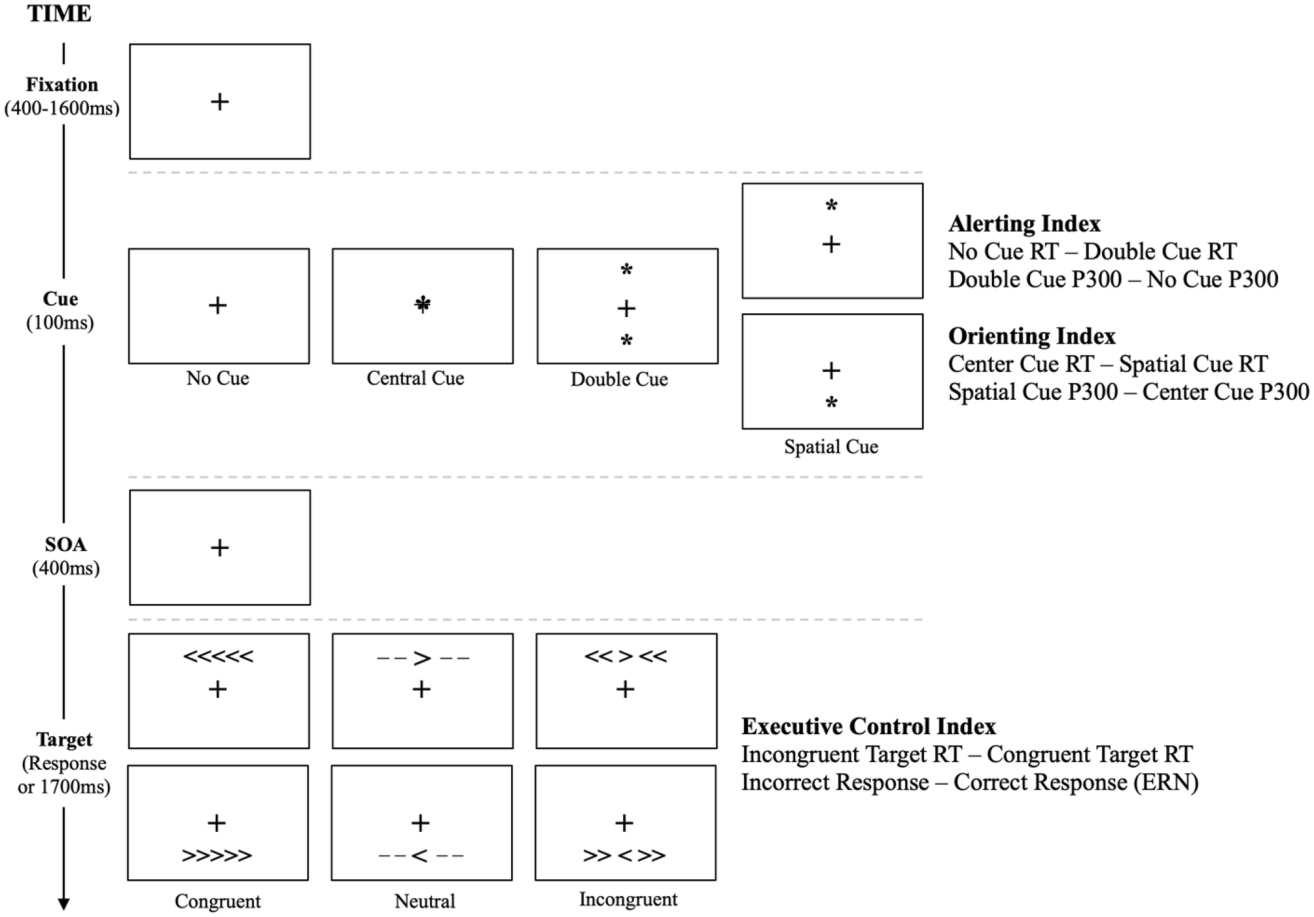
The attention network task.

Behavioral and neural metrics of alerting, orienting, and executive control were derived from the Attention Network Task (Fig. 4)^32^.

The ANT combines temporal and spatial cueing to assess the alerting and orienting attention networks^33^ with a flanker congruency paradigm to assess executive control^34^. Reaction times (RTs) to the target stimuli are used to quantify the processing efficiency of each network. For example, the difference in RT to the target stimuli following no cue compared to the double cue indicates the processing efficiency of the alerting network, the difference in RT to the target stimuli following the center cue compared to the spatial cue indicates the processing efficiency of the orienting network, and the difference in RT to the incongruent compared to the congruent targets indicates the processing efficiency of the executive control network^32^.

In addition to behavioral metrics, we make a novel contribution to the Attention Restoration Theory literature by time-locking event-related potentials (ERPs) to the ANT, providing an additional level of analysis of alerting, orienting, and executive control. Consistent with prior work in other fields^54,55^, neural metrics of alerting and orienting can be assessed by quantifying P300 amplitude generated by the different cue trials of the ANT. The P300 is a positive deflection in the brain's electrical activity that typically occurs around 300 ms after stimulus presentation. The P300 reflects the brain's response to unexpected or infrequent stimuli, illustrating its role in alerting and orienting the individual to novel or salient events in their environment and facilitating their ability to shift and maintain attention accordingly^56^. The P300 is thought to be generated by a network of neural structures throughout the brain that changes depending on the task at hand. For relevance to this study, the P300 is maximal over posterior parietal cortices when an individual completes a task that engages alerting and orienting of attention^57^. This cortical region plays a crucial role in maintaining an alert state, directing attention to spatial locations, and integrating sensory information with motor information to guide behavior^58^.

The executive control function of attention can be assessed by quantifying error-related negativity (ERN) amplitude generated by correct and incorrect responses to each target. The ERN is a negative deflection in the brain's electrical activity that occurs shortly after an individual commits an error on a task. Thus, the ERN reflects the brain's ability to detect and evaluate errors in real-time, which is a crucial aspect of cognitive control and self-regulation. This component plays a role in the executive control process by signaling the need for adjustments in attention and behavior to optimize task performance and minimize errors^59^. Source-localization studies have consistently demonstrated that the ERN is generated in the anterior cingulate cortex^60–63^, an integrative hub in the brain that is highly involved in executive control^64^.

Like the RT formulas, ERP metrics were calculated by comparing target responses under the following cue conditions: double cue minus no cue (alerting), spatial cue minus center cue (orienting), and incorrect minus correct responses (executive control). These cue-network relationships and the outcome measures used to assess them are summarized in Table 3.

**Table 3:** Summary of the behavioral and neural ANT metrics used to assess the three functions of attention.

Attention network	Level of analysis	Dependent variable	Factor
Alerting	Behavioral Neural	RT P300 amplitude	Cue condition (no cue-double) Cue condition (double-no cue)
Orienting	Behavioral Neural	RT P300 amplitude	Cue condition (center-spatial) Cue condition (spatial-center)
Executive control	Behavioral Neural	RT ERN amplitude	Target condition (incongruent—congruent) Response Type (incorrect–correct)

### Stimulus presentation

Self-report surveys (i.e., demographic questionnaire, depletion manipulation check, and the Perceived Restorativeness Scale) were administered via Qualtrics (Qualtrics, Provo, UT) on a 5th generation iPad mini. The Attention Network Task was programmed in PsychoPy v3.1.3 and presented to participants on a monitor positioned 18 inches from their head. At each time point (pre-walk and post-walk), participants completed three blocks of 192 trials of the ANT, with self-paced breaks between each block. The task took about 18 min to complete.

All target stimuli were presented in black ink on a gray background and the target stimuli that participants responded to subtended 2.60 degrees of visual angle. In each trial, a fixation cross (400–1600 ms randomized ISI) was followed by one of four cue conditions (no cue, central cue, double cue, spatial cue) that appeared on the screen for 100 ms. After cue presentation, the fixation cross appeared on the screen again for 400 ms followed by one of three target conditions (congruent, incongruent, or neutral) consistent with the flanker congruency paradigm^34^. The target condition remained on the screen until the participant responded, or until 1700 ms had passed (see Fig. 4). Participants responded to the direction of the arrow in the middle of the target stimulus using the arrow keys on the keyboard and their right hand.

### EEG recording and processing

EEG data were collected with a 32-channel, active electrode actiCap manufactured by BrainVision (BrainVision Systems, Morrisville, NC, USA). Channel locations were pre-determined according to the International 10–20 system^65^ and all impedances were kept below 25 kOhms. The signal was amplified with the BrainVision actiCHamp Plus amplifier with an online sampling rate of 500 Hz and acquired by BrainVision Recorder (Version 1.20.0601). The online and offline reference channel was the right mastoid (TP10). Two auxiliary electrodes were placed above and below the right eye to record blinks and eye movements for later processing.

EEG data were processed in MATLAB using the EEGLab^66^ and ERPLab^67^ toolboxes. Raw data were downsampled to 250 Hz and band pass filtered from 0.1 to 30 Hz. Artifacts created by blinks and eye movements were corrected for using Gratton’s Eye Movement Correction Procedure (EMCP)^68^. To account for any artifacts left uncorrected by EMCP, additional artifact rejection was performed using a moving window to reject sections of data containing flatlines or peak-to-peak activity greater than 200 μV^67^.

#### P300

The P300 component was used to quantify alerting and orienting on a neural level^56^. To quantify the stimulus-locked P300, continuous data were epoched from 700 ms before target presentation to 700 ms after target presentation, with the average activity from – 700 to − 500 ms pre-target presentation (i.e., − 300 to − 100 ms pre-cue presentation) serving as the baseline^55^. A measurement window from 250 to 450 ms after target onset was used for the alerting and orienting analysis^54^.

To assess the alerting and orienting facets of attention, the stimulus-locked P300 was binned and averaged separately by cue type (no cue, double cue, center cue, spatial cue) and target type (congruent, incongruent, and neutral). Consistent with Fan et al. (2002)’s formulae^32^, we subtracted the no cue P300 waveform from the double cue P300 waveform (Double Cue-No Cue) to create the P300 for the alerting network. To create the P300 for the orienting network, we subtracted the center cue P300 waveform from the spatial cue P300 waveform (Spatial Cue–Center Cue). We then quantified these P300 difference waveforms by extracting the mean amplitude within the 250–450 ms measurement window for each facet of attention using the ERP Measurement Tool in the ERPLab toolbox^67^. The P300 was analyzed at electrode Pz, as this is where it was maximally seen in this study.

The average percent of epochs lost in the P300 analysis after artifact handling was 3.33% (Nature Pre-walk ANT: 2.50%, Nature Post-walk ANT: 4.94%, Urban Pre-walk ANT: 1.32%, Urban Post-walk ANT: 4.60%).

#### Error-related negativity

The ERN component was used to quantify executive control on a neural level^59^. Consistent with all ERN research that utilizes a flanker congruency paradigm, the ERN was time-locked to the participants’ response to the flanker arrow presentations^34^. To quantify the ERN, continuous data were epoched from − 400 ms pre-response to 400 ms after response. The average activity in the -400 to -200 ms pre-response window served as the baseline to avoid baselining into any pre-motor response brain activity. A measurement window from 7 to 57 ms after response was determined using the collapsed localizer technique^69^. This technique involves creating the grand average waveform (collapsed across all participants, timepoints, cue types, and target types) and using this aggregate waveform to identify the peak latency of the overall ERN difference wave (Incorrect–Correct). The collapsed localizer technique revealed an average ERN peak latency of 32 ms. The measurement window for the ERN was ± 25 ms around the peak (i.e., 7–57 ms).

To assess the executive control facet of attention, the response-locked ERN was binned and averaged separately by response type (incorrect and correct) and the processing efficiency of the executive control network was calculated by creating a difference wave by subtracting the correct waveform from the incorrect waveform (Incorrect–Correct). We quantified the ERN waveforms by extracting the mean amplitude from 7 to 57 ms post-response for each participant at each timepoint using the ERP Measurement Tool in the ERPLab toolbox^67^. The ERN was quantified at electrode Cz, as this is where it was maximally seen in this study.

Consistent with prior ERN research, a conservative error cutoff was employed to ensure reliable ERN amplitudes, therefore participants that made 7 or fewer errors on the ANT after artifact rejection were not included in the ERN analysis^70^, which resulted in the exclusion of 18 out of the 184 ANT files (8 nature pre-walk files, 6 nature post-walk files, 3 urban pre-walk file, and 1 urban post-walk file). The average percent of epochs lost in the ERN analysis after artifact handling was 1.60% (Nature Pre-walk: 1.56%, Nature Post-walk: 2.23%, Urban Pre-walk: 0.74%, Urban Post- walk: 1.95%).

### Statistical analyses

All analyses were conducted in R version 4.0.2^71^. To compare depletion manipulation check and Perceived Restorativeness Scale results between the two walking groups, independent samples t-tests were run using the ‘t.test’ function in R. For all ANT results, linear mixed effect models using the ‘lmer’ function in R’s ‘lme4’ package^72^ were utilized to control for sources of non-independence in the data (i.e., repeated-measures within an individual) and allow for missing data (e.g., if a participant was missing data from a single time point). All models included the behavioral or neural ANT metric as the dependent variable, Participant as a random intercept, and the interaction between Time (Pre-walk versus Post-walk) and Condition (Nature versus Urban) as a fixed effect predictor to determine if any pre- to post-walk changes differed between the nature and the urban group. To test the significance of all fixed effects we ran likelihood ratio tests using the ‘anova’ function in the ‘stats’ package. These tests generated a chi-squared statistic comparing the model with the variable of interest (Time, Condition, and the Time by Condition interaction) entered as a fixed effect and Participant entered as a random intercept, to a model with the fixed effect of interest removed. We calculated Cohen’s *d* effect sizes for all significant effects.

## Results

One nature participant’s post-walk data were not included in any analyses because their testing session was cut short due to rain and they were unable to complete their walk.

### Depletion manipulation check

**Figure 5:**
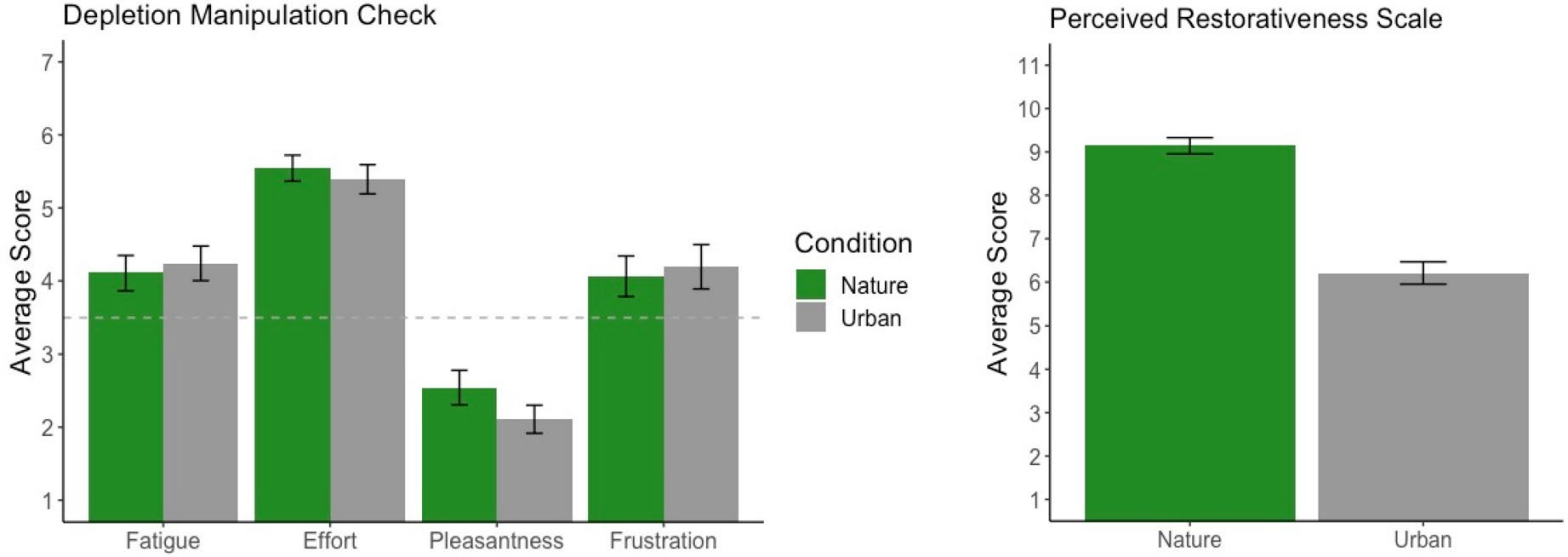
Self-report results. Left: results from the four depletion manipulation check questions. The horizontal dotted line represents the median rating (3.5) for each question. Right: results from the PRS-11. Error bars represent one standard error of the mean.

Four manipulation check questions related to fatigue, effort, pleasantness, and frustration were administered immediately following the depletion task. These manipulation check questions ensured that participants felt depleted after counting backwards and that reported levels of depletion were comparable across groups. Results of the manipulation check can be visualized in Fig. 5. To ensure the integrity of the counting backwards depletion task, we confirmed that, on average, participants rated the pleasantness of the task as below the median rating (below a 3.5 on the 7-point Likert-scale) and their levels of fatigue, effort, and frustration above the median rating (above a 3.5 on the 7-point Likert-scale). To ensure that each group was comparably depleted before their walk, we compared group means for each question on the depletion manipulation check survey using independent samples t-tests. There were no significant group differences in self-reported fatigue (*t*(89.99) = -0.39, *p* = 0.700), mental effort (*t*(88.80) = 0.57, *p* = 0.571), pleasantness (*t*(86.44) = 1.43, *p* = 0.157), or frustration (*t*(89.30) = -0.32, *p* = 0.750).

### Perceived Restorativeness Scale (PRS-11)

At the end of testing, participants rated how restorative they perceived the environment they walked in to be. Results from the 11-item scale can be visualized in Fig. 5. As expected, the natural environment (*M* = 9.14, *SD* = 1.27) was perceived as significantly more restorative than the urban environment (*M* = 6.21, *SD* = 1.74; *t*(80.51) = 9.11, *p* < 0.001, 95% CI [2.29 3.57], Cohen’s *d* = 1.92).

### Alerting

**Figure 6:**
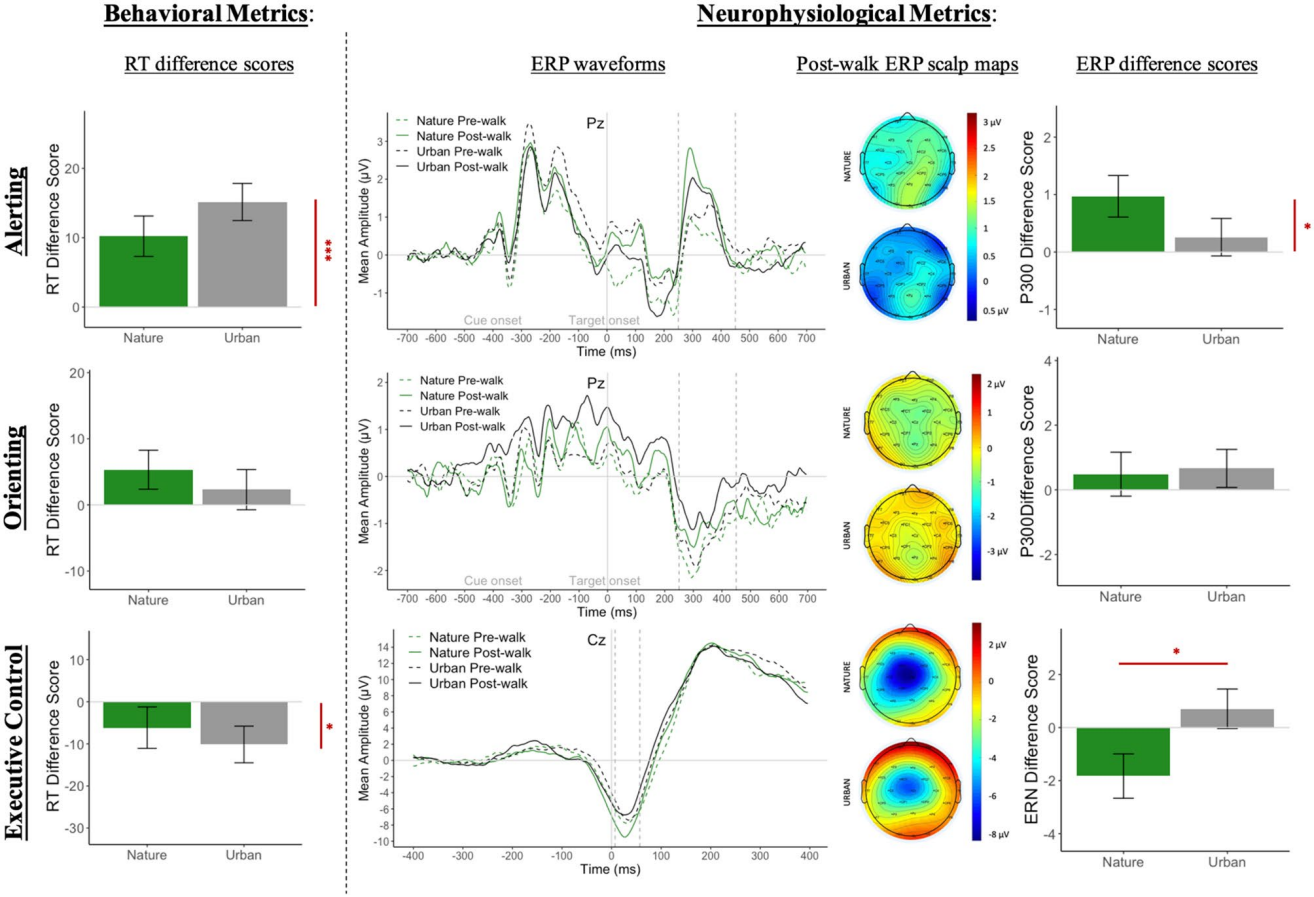
Attention network task results. The behavioral metrics include RT index difference scores (post-walk minus pre-walk) for each group. The neurophysiological metrics include the ERP waveforms for each ANT index, post-walk scalp maps of the associated ERP component for each group, and difference scores (post-walk minus pre-walk) of the associated ERP component for each group. Error bars represent one standard error of the mean of the difference scores. Y-axes are standardized to 1.5 SD around the grand mean per recommendation from Witt (2019)^73^.

The alerting facet of attention was quantified behaviorally (RT) and neurophysiologically (P300). Descriptive statistics of these two alerting indices as a function of Time and Condition can be seen in Supplemental Materials and pre- to post-walk difference scores can be visualized in Fig. 6. Increases in RT and P300 alerting indices indicate an improvement in alerting.

Behaviorally, linear mixed models revealed a main effect of Time on the RT alerting index (χ^2^(1) = 31.81, *p* < 0.001) such that there was a significant increase in the RT alerting index from pre-walk to post-walk (β = 12.39, *SE* = 2.00, *df* = 88.48, *t* = 6.19, *p* < 0.001, 95% CI [8.43 16.31], Cohen’s *d* = 0.64). There was no significant main effect of Condition on the RT alerting index (χ^2^(1) = 1.57, *p* = 0.210), nor was there a significant Time by Condition interaction (χ^2^(1) = 1.30, *p* = 0.254). Neurophysiologically, there was a significant main effect of Time on P300 alerting index (χ^2^(1) = 6.18, *p* = 0.0129) such that mean amplitude increased from pre-walk to post-walk (β = 0.62, *SE* = 0.25, *df* = 90.75, *t* = 2.51, *p* = 0.0138, 95% CI [0.13 1.10], Cohen’s *d* = 0.28). There was no significant main effect of Condition on P300 alerting index (χ^2^(1) = 0.01, *p* = 0.917), nor was there a significant Time by Condition interaction (χ^2^(1) = 2.23, *p* = 0.137). There was no significant correlation between the RT alerting index and the P300 alerting index (Pearson’s *r* = 0.12, *p* = 0.270).

### Orienting

The efficiency of the orienting network was quantified behaviorally (RT) and neurophysiologically (P300). Descriptive statistics of these two orienting indices as a function of Time and Condition can be seen in Supplemental Materials and pre- to post-walk difference scores can be visualized in Fig. 6.

Behaviorally, linear mixed models revealed no significant main effect of Time (χ^2^(1) = 2.94, *p* = 0.087), no significant main effect of Condition (χ^2^(1) = 0.67, *p* = 0.414), and no significant Time by Condition interaction (χ^2^(1) = 0.57, *p* = 0.449) on the RT orienting index. Neurophysiologically, models show similar null results of Time (χ^2^(1) = 1.67, *p* = 0.196), Condition (χ^2^(1) = 0.40, *p* = 0.528), and Time by Condition interaction (χ^2^(1) = 0.055, *p* = 0.816) on the P300 orienting index. There was no significant correlation between the RT orienting index and the P300 orienting index (Pearson’s *r* = 0.14, *p* = 0.197).

### Executive control

The efficiency of the executive control network was quantified behaviorally (RT) and neurophysiologically (ERN). Descriptive statistics of these two executive control indices as a function of Time and Condition can be seen in Supplemental Materials and pre- to post-walk difference scores can be visualized in Fig. 6. Decreases in RT and ERN executive control indices indicate an improvement in executive control.

Behaviorally, linear mixed effects models revealed a main effect of Time on the RT executive control index (χ^2^(1) = 5.90, *p* = 0.015) such that there was a significant decrease in RT executive control index from pre-walk to post-walk (β = − 8.07, *SE* = 3.28, *df* = 89.88, *t* = − 2.46, *p* < 0.05, 95% CI [− 14.53 − 1.59], Cohen’s *d* = − 0.19). There was no significant main effect of Condition (χ^2^(1) = 0.17, *p* = 0.684), nor a significant Time by Condition interaction (χ^2^(1) = 0.358, *p* = 0.549) on the RT executive control index. Neurophysiologically, there was no significant main effect of Time (χ^2^(1) = 0.28, *p* = 0.600) or Condition (χ^2^(1) = 1.32, *p* = 0.251) on the ERN executive control index. There was, however, a significant interaction between Time and Condition (χ^2^(1) = 4.47, *p* = 0.034) such that nature walkers showed a more enhanced ERN from pre- to post-walk (indicating an improvement in executive control) than urban walkers did (β = 2.39, *SE* = 1.13, *df* = 79.20, *t* = 2.12, *p* = 0.0368, 95% CI [0.18 4.59], Cohen’s *d* = 0.41). There was a significant positive correlation between the RT executive control index and the ERN executive control index (Pearson’s *r* = 0.30, *p* = 0.0083), such that an increase in the RT executive control index was associated with an increase in mean amplitude of the ERN.

### Relationship to perceived restorativeness

Participants’ average score on the PRS-11 did not correlate with changes in either alerting index, either orienting index, or the RT executive control index. Interestingly though, average score on the PRS-11 did significantly correlate with change in ERN amplitude from pre- to post-walk (Pearson’s *r* = − 0.26, *p* = 0.0247) such that the more restorative a participant rated their walk to be, the larger their ERN. A series of regression models were run to better understand the relationship between self-reported perceived restorativeness, walk condition, and change (post-walk minus pre-walk) in ERN amplitude. There was a main effect of perceived restorativeness on change in ERN amplitude (β = − 0.61, *SE* = 0.27, *t* = − 2.29, *p* = 0.0247, 95% CI [− 1.15 − 0.080], Cohen’s *d* = − 0.12) and a main effect of Condition on change in ERN amplitude (β = 2.54, *SE* = 1.14, *t* = 2.23, *p* = 0.0289, 95% CI [0.27 4.81], Cohen’s *d* = 0.49). However, the interaction between perceived restorativeness and Condition was not statistically significant (β = 0.58, *p* = 0.507, 95% CI [− 1.15 2.32]), indicating that the relationship between perceived restorativeness and change in ERN amplitude was consistent across both groups.

## Discussion

This study took a rigorous experimental approach to assessing the influence of immersion in nature on attention-processes in the brain. We assessed three facets of attention (alerting, orienting, and executive control), from multiple levels of analysis (behavioral and neural), with a sample size that exceeds prior experimental studies in the Attention Restoration Theory literature. We set out to answer which aspects of attention, if any, are influenced by a short-term (40-min) immersion in nature.

To experimentally recreate Kaplan’s idea of the depleted cognitive state from which individuals may seek out nature, participants underwent a depletion protocol to ensure they were ‘primed’ for maximal restoration potential. Participants were randomized to either a 40-min walk in nature or a 40-min walk in a control, urban environment of comparable distance. The two walking routes utilized in this study were designed in accordance with the four qualifications of a restorative environment outlined in Attention Restoration Theory^15^. It was theorized that a 40-min immersion would be long enough to meet the qualification of *being away*, but also short enough to still be *compatible* with most people. The natural environment used in the experiment had substantial *extent* and depth of processing, as participants walked along a trickling creek, through an oak tree tunnel, and around a pond with ducks and a small waterfall. It is assumed that these features engaged the senses in the ‘*softly fascinating*’ way proposed by Attention Restoration Theory. As expected, participants reported that the natural environment was, indeed, significantly more restorative than the urban environment, as measured by the Perceived Restorativeness Scale (PRS-11).

To assess each aspect of attention within a single cognitive task, participants completed the Attention Network Task (ANT) before and after their walk. The ANT can elucidate whether the attentional benefits of exposure to nature are specific to a particular aspect of attention or if they encompass all three dimensions in a domain-general manner. Notably, this study is the first in the Attention Restoration Theory literature to synchronize ERPs with the ANT, allowing for the dual examination of task performance from a behavioral and neurophysiological standpoint. This simultaneous co-registration of behavioral and neural data not only facilitates the assessment of task performance but also yields insights into the underlying neural mechanisms engaged during the task. Consequently, this approach yields a more comprehensive understanding of potential cognitive changes in response to immersion in nature, shedding light on the intricate interplay between nature exposure and cognitive functioning. We hypothesized that nature exposure would improve the executive control indices of the ANT, as executive control is thought to align closest with the construct of directed attention described in Attention Restoration Theory^18^. We predicted that there would be no changes in alerting or orienting.

Contrary to our hypothesis, results suggest that *both* groups showed enhanced alerting after the walk, denoted behaviorally by an increase in the RT alerting index and neurophysiologically as an increase in the P300 alerting index from pre- to post-walk. It can be concluded that 40-min (~ 2-miles) of low-intensity walking may improve alerting on both a behavioral level and a neural level regardless of the type of environment one walked in. This is supported in the exercise and cognition literature, which shows that low- to moderate-intensity exercise enhances the alerting index on the ANT^54^. The low-intensity walk utilized in this study likely enhanced arousal and freed up available attentional resources, reflected by an increase in RT and P300 alerting indices for both groups.

In terms of orienting attention, our hypothesis was supported in that neither group showed changes in either the behavioral or neural index of orienting. This suggests that neither immersion in nature nor even exercise influence orienting efficiency. This is likely because orienting is not a particularly effortful facet of attention and is thus less sensitive to being depleted in the first place.

We are particularly interested in where environment-specific effects diverge from general exercise effects—namely, where there is a distinction between the nature and urban group. We see this differentiation in the executive control results. In line with our hypothesis that there would be an improvement in executive control associated with immersion in nature, nature walkers showed enhanced (more negative) ERN amplitude after their walk while urban walkers showed no significant changes in ERN amplitude. This replicates prior findings of enhanced ERN amplitude during a 4-day immersion in nature compared to immersion in a control, urban environment^51^. The ERN occurs within the first 100 ms following the commission of an error on the ANT, indexing error detection and performance monitoring in the brain^74^. The larger the ERN response, the more likely an individual is actively monitoring their performance and adjusting their behavior to reduce future errors. This is a key aspect of executive control. Through the framework of Attention Restoration Theory, this suggests that nature likely allowed the neural mechanisms related to executive control to rest and recuperate, leading to increased executive control capacity (illustrated by an increased ERN) when tasked with completing the ANT after the walk. This conclusion is further supported by the observed significant relationship between score on the PRS-11 and change in ERN amplitude, suggesting that the changes in ERN amplitude are, at least to some degree, associated with restoration. However, this relationship should be interpreted with caution given the effect size is small (Cohen’s *d* = − 0.12) and there is no significant interaction between perceived restorativeness and condition in predicting change in ERN amplitude. Future work should further disentangle the relationship between the ERN, self-reported perceived restoration, and environment type.

The ERN is generated in the anterior cingulate cortex, a subcortical structure the brain that plays a crucial role in integrating cognitive, emotional, and motivational information from the environment to guide subsequent behavior^75,76^. Importantly, the anterior cingulate cortex is functionally connected to the prefrontal cortex during tasks that engage aspects of executive control such as error detection and response inhibition—processes that are assessed in this study by the executive control portion of the ANT. Perhaps the decrease in attentional demands present in natural environments allows for anterior cingulate-prefrontal cortex functional network to rest and recover, allowing for enhanced functionality when required to come online during ANT completion after the walk. This suggests a potential underlying neural mechanism for executive control restoration in nature that should be expanded upon in future work.

It is interesting to note that the RT executive control index and the ERN executive control index show different patterns such that both groups improved on a behavioral level but only the nature group improved on a neural level. The lack of a behavioral effect is consistent with some prior work that fails to find differences in the RT executive control index on the ANT between a nature walk and an urban walk^38–40^. The dissociation between behavioral and neural results presented in this study illustrates the strength of utilizing multiple dependent measures when assessing cognition, suggesting that different dependent measures have different sensitivity to experimental manipulations. It is not uncommon to see this play out in the cognitive neuroscience literature at large. For one, neurophysiological metrics may detect subtler, possibly pre-conscious processes, while behavioral metrics capture more integrated and conscious responses^45^. Additionally, neurophysiological measures may detect changes that are not large enough to affect behavior, which often requires a change to pass a certain threshold before it is reflected in task performance. Nevertheless, such patterns indicate a complex and nuanced relationship between brain activity and behavior.

In this study, we can only speculate as to why we see this dissociation between the behavioral and neural results. For one, it is possible that because the RT executive control index is calculated from just the correct trials and the ERN is calculated based on error trials, they are capturing different aspects of executive control, with some aspects of executive control being more strongly influenced by immersion in nature than others. The RT executive control index taps into inhibitory control processes (ignoring distracting flanker arrows to respond quickly and accurately to the central target), whereas the ERN indexes error processing and performance monitoring (recognizing and responding to an error). While our results show a significant correlation between the RT executive control index and the ERN executive control index (Pearson’s *r* = 0.30), this correlation is small, accounting for only 9% of the variance. Therefore, while both processes reflect aspects of executive control, they are dissociable constructs that serve different roles in how an individual attends to their environment. Interestingly, there is prior literature that similarly fails to find nature-related improvements in inhibitory control processes such as on the Stroop Task^77^ and literature that does find improvements in performance monitoring^51,78^. This suggests that perhaps not all forms of executive control are comparably influenced by exposure to nature. Again, these divergent results accentuate the strength of co-registering behavioral with neurophysiological metrics and utilizing multiple dependent measures in research, as it allows for deeper insight into cognition than any single measure can provide on its own. Nevertheless, future work should collect behavioral and neural measures simultaneously to see if the pattern holds.

This study was designed specifically to test which aspects of attention, if any, are restored following a 40-min nature compared to urban walk. As hypothesized, we found that the nature walk, but not the urban walk, enhanced amplitude of the ERN—a neural index of the executive control aspect of attention. Our research question and hypotheses were based on extant theory and prior literature that has validated the propositions set forth by Attention Restoration Theory. Based on this robust body of prior work, as well as participant reports of greater perceived restoration after the nature walk compared to the urban walk, we believe we are, at least in part, observing attention restoration effects of immersion in nature. As is unavoidable when exploring the impact of immersion in real-world environments on cognition, there are inevitably several uncontrollable variables that may influence results. To control for some of these, we randomly assigned participants to conditions and carefully designed our two walking routes to be comparable in distance, elevation change, amount of time outside, and pace (see Table 2). Additionally, our two groups were well-matched on exercise metrics such as average heart rate and calories burned. Furthermore, in a series of exploratory analyses presented in Supplemental Materials , we statistically ruled out other potential variables that may influence our ERN results, such as age, temperature, and time of day. However, it is still possible that there are other, unaccounted for variables at play that may be influencing the observed enhanced ERN. For instance, it is possible that there are group differences in stress. Roger Ulrich’s Stress Recovery Theory complements Attention Restoration Theory in that it posits that natural environments are restorative because they aid in recovery from stress^79^. While this study did not directly measure or manipulate stress, it is likely that there is an element of stress recovery co-occurring alongside attention restoration, an idea that has been proposed in a recent unified framework of restoration in nature^80^. This study was rigorous in controlling for many variables; however, the results should be interpreted with caution, given there may still be unidentified factors influencing ERN amplitude beyond attention restoration.

What constitutes “natural” and “urban” environments exists on a spectrum from untouched wilderness to concrete jungle. Thus, the ideal walking environments would perhaps be a nature walk in a remote wilderness area and an urban walk in a highly-congested downtown neighborhood. Given the logistical constraints of the current study, we were limited in the type of environments to choose from. This constraint left us with a protected natural environment with some “built” characteristics such as paved walkways and bridges. This arboretum was adjacent to an urban environment that had some trees lining the sidewalk and a distant view of mountains. While these environments are not at the polar ends of the spectrum described above, they do accurately represent environments that are realistically accessible to most individuals on a day-to-day basis, increasing the ecological validity of this work.

Additionally, given certain outcome measures improved in both walking groups (namely, the two alerting metrics and the behavioral executive control metric), the data suggest that exercise, in and of itself, improves certain aspects of attention regardless of the environment. The methodological decision to have participants walk in these environments rather than just sit in them may be perceived as a limitation of this work. We made this decision because we felt it was important for participants to engage and interact with their environments in a way that would not have been possible just sitting. This was in line with Attention Restoration Theory’s criterion for an environment to have *extent* to be restorative. This decision was also based on the substantial body of prior studies that require participants to walk in nature rather than sit in nature and still find differential effects between natural and urban environments^35,38,44^. Lastly, because individuals often exercise when they are in natural settings (e.g., a walk in a park, trail running, mountain biking, skiing), we felt it would be important for the ecological validity of the work to incorporate this to see if nature differentially influences attention above and beyond the effects of just exercise. However, future work could replicate this protocol but have participants sit in each environment for 40-min rather than walk in them or employ a direct manipulation of exercise in nature.

This study demonstrates an initial exploration into understanding the underlying neural mechanisms of nature’s attentional benefits. Future work should seek to answer what the *optimal* dosage of nature is to realize these benefits (e.g., minutes, hours, or days), how different *types* of nature may differentially impact cognition (e.g., ocean, forest, mountains, desert), and how long these effects last. In the present study, participants completed their second ANT within 20 min of their walk. It is possible that the observed enhancements in executive control may be a fleeting benefit that quickly dissipates as one re-enters their typical urban environment. Furthermore, an interesting and important step in the field will be to delineate the restorativeness of nature from the restorativeness of being away from technology. Participants in this study were instructed to leave their cellphones in the laboratory so that it would not interfere with the restorative potential of their walk. Therefore, it is possible that some of the benefits we see in nature may be driven by separation from technology. Anecdotal evidence would suggest that being immersed in nature without cellphone service or the ability to refresh an email inbox is more restorative than being outside and still connected to work and social media. However, this needs to be directly tested. Jiang et al. (2019)^14^ experimentally manipulated technology use during a 15-min immersion in nature and found that participants who were in nature on a laptop did not receive the same cognitive benefits as those without technology^14^. Future work can replicate this type of design using neurophysiological measures to see if engaging with technology disrupts the neural changes we have identified.

While beyond the scope of the present study, this field of environmental neuroscience holds implications for environmental policy, clinical psychology, and urban design. Compared to studies based exclusively on self-report and behavioral measures, studies that utilize cognitive neuroscience methods may be more successful in influencing government action and public concern for the health of the environment. As Roger Ulrich—a seminal figure in environmental psychology—suggests, research employing neurophysiological measures carries more weight than behavioral or subjective measures in political decision-making and will therefore be more effective in the implementation of environmentalist policy^42^. Moreover, studies suggest that nature-based interventions may be effective in improving health outcomes across a variety of conditions, such as attention-related disorders or depression^81^. Lastly, understanding the relationship between physical environments and the individual can also contribute to urban design geared toward optimizing human functioning. Perhaps the location and design aesthetics of buildings such as workplaces, hospitals, and prisons will prioritize exposure to nature. Work schedules that allow for more time outside and less time in front of a computer screen could optimize cognitive performance and productivity of employees.

In sum, this well-powered, tightly-controlled study sought to improve our understanding of the effects of a nature walk on the brain and behavior. It employed sophisticated electrophysiological techniques to offer intriguing results that a walk in nature enhances certain executive control processes in the brain above and beyond the benefits associated with exercise. This study demonstrates the power of utilizing EEG to uncover the benefits of nature on human cognition and provides a framework for future work to explore which *types* of nature enhance executive control and how *much* time in nature is needed to do so.

### Supplementary Information

Supplementary Information.

## Data Availability

De-identified data as well as MATLAB and R code for data processing and analysis are available on OSF at https://osf.io/px4ev/?view_only=ec6ec86bd497482795d821386543d802.
