# Supplementary material for: Immersion in nature enhances neural indices of executive attention
Source: Sci Rep. 2024 Jan 22;14:1845. doi: 10.1038/s41598-024-52205-1 (PMC10803324; doi:10.1038/s41598-024-52205-1)
Supplement: Supplementary file 1 — Supplementary Information. [file 41598_2024_52205_MOESM1_ESM.docx]

**SUPPLEMENTAL MATERIALS FOR:**

**Immersion in nature enhances neural indices of executive attention**

Amy S. McDonnell^1^ & David L. Strayer^1^

^1^Department of Psychology, University of Utah, Salt Lake City, UT, 84102, USA

**SUPPLEMENTAL MATERIALS**

**Depletion Manipulation Check Questions**

The following questions were adopted from Bray et al. (2008) and were administered to each participant after they completed the counting backwards depletion task:

Please answer these four questions on a scale of 1 (not at all) to 7 (extremely).

____1. How tired do you feel after completing this task?

____2. How much effort did you exert while performing this task?

____3. How pleasant did you find performing this task?

____4. How frustrated did you feel while performing this task?

**Perceived Restorativeness Scale (PRS-11)**

The following scale (developed by Pasini et al., 2014) was administered to each participant at the end of the experimental protocol to measure the restorative quality of the two environment types:

We are interested in how you experienced your walk today. To help us understand your experience, we have provided the following statements for you to respond to. Please read carefully, then ask yourself: "how much does this statement apply to the environment I took my walk in today?".

To indicate your answer, select one of the numbers on the rating scale below each statement. For example, if you think that the statement does not at all apply to your experience of the environment you walked in, then you would select "0" (not at all), if you think it applies rather much, then you would select "6" (rather much), but if you think that it applies very much, you would select “10” (completely).

_____ 1. Places like that are fascinating.

_____ 2. In places like this my attention is drawn to many interesting things.

_____ 3. In places like this it is hard to be bored.

_____ 4. Places like that are a refuge from nuisances.

_____ 5. To get away from things that usually demand my attention I like to go to places

like this.

_____ 6. To stop thinking about the things that I must get done I like to go to places like

this.

_____ 7. There is a clear order in the physical arrangement of places like this.

_____ 8. In places like this it is easy to see how things are organized.

_____ 9. In places like this everything seems to have its proper place.

_____ 10. That place is large enough to allow exploration in many directions.

_____ 11. In places like that there are few boundaries to limit my possibility for moving

about.

**Video Recording and Reduction**

Participants were outfitted with a GoPro HERO 7 video camera (GoPro, Inc.) secured via a chest strap (see Figure 3 in main text). Videos were recorded with 1080p resolution, at 60 frames per second, with a linear field of view and HyperSmooth video stabilization. Video footage was collected for an added layer of quality control, given participants walked alone and were not accompanied by the researcher. Reviewing the footage allowed the research team to identify any events of concern that may have happened on the walk that would have deemed that participant’s data unusable (e.g., diverting too far from the route, having a negative interaction with a passerby, getting injured, etc.). Importantly, after these thorough video inspection and reduction protocols, there was no concerning footage that led to the exclusion of any participants’ data.

Meaningful events, behaviors, and environmental characteristics for each of the two walking conditions were recorded using this naturalistic video data. Raw video files for each participant were uploaded to iMovie (version 10.1.12), stitched together into a single, ~40-minute video file for each participant, and then uploaded to Behavioral Observational Research Interactive Software (BORIS; version 7.13.9; Friard & Gamba, 2016)—an event-logging software developed for coding live video. Behaviors and events that the research team deemed meaningful were logged and coded into the following event categories: Person, Group, Vehicle, Bicycle, Map, and GPS watch. For definitions of each of these events, see Table S2. Coded data were exported for each participant and compiled into one large dataset from which descriptive statistics were averaged across all participants and between walking conditions. To view descriptive statistics of events in each of the walk conditions as captured by the video recordings, see Table S3.

Table S1 Definitions of events and behaviors coded from the naturalistic data.

| **Event/Behavior** | **Definition** |
| --- | --- |
| **Person (#)** | Participant walked past a person on foot |
| **Group (#)** | Participant walked past a group of eight or more people (e.g., tour group on the urban route or yoga class on the nature route) |
| **Vehicle (#)** | Motorized vehicle drove past the participant |
| **Bicycle (#)** | Participant walked past a person on a bicycle |
| **Map (#)** | Participant directed their attention toward their map |
| **GPS watch (#)** | Participant checked their GPS watch (e.g., to monitor their pace) |

Table S2. Descriptive statistics of each event/behavior as a function of walk condition.

|  | **Urban condition** | | **Nature condition** | |
| --- | --- | --- | --- | --- |
|  | **Mean** | **SD** | **Mean** | **SD** |
| **Person (#)** | 8.46 | 4.49 | 28.38 | 20.64 |
| **Group (#)** | 0.04 | 0.21 | 1.60 | 2.64 |
| **Vehicle (#)** | 153.43 | 65.90 | 1.49 | 1.60 |
| **Bicycle (#)** | 5.74 | 3.19 | 0.07 | 0.25 |
| **Map (#)** | 5.96 | 7.80 | 20.71 | 14.67 |
| **GPS watch (#)** | 16.98 | 14.98 | 11.51 | 10.26 |

**Attention Network Task Descriptive Statistics**

The Attention Network Task (Fan et al., 2002) was utilized to assess the efficiency of each attention network (alerting, orienting, and executive control) before and after either a walk in an urban environment or a walk in a natural environment. Behavioral metrics (reaction time and error rate) as a function of target type and cue type can be visualized in Figure S1 and Table S3, which serves as a manipulation check that the task outcomes are consistent with prior literature (e.g., that reaction times and error rates are highest to incongruent stimuli). A summary of descriptive statistics of each ANT index as a function of Time (Pre-walk versus Post-walk) and Condition (Nature versus Urban) can be seen in Table S4.


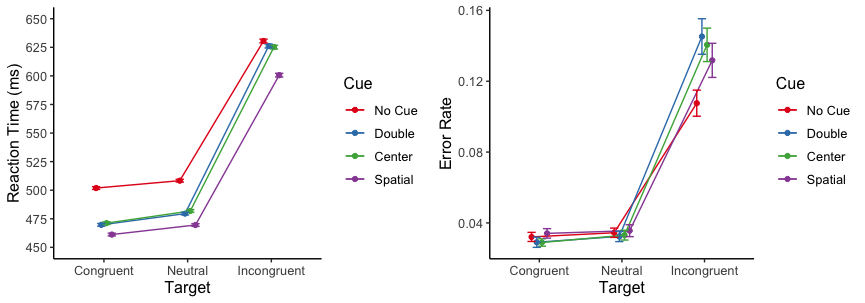


Figure S1. Behavioral data with reaction time (left) and error rate (right) stratified by cue and target type. Error rates are out of 1.0. Error bars represent standard error of the mean.

Table S3. Means and standard errors of RT (ms) and Error Rate as a function of cue type and target type.

| **Target Type** | **Cue Type** | | | |
| --- | --- | --- | --- | --- |
|  | **None** | **Center** | **Double** | **Spatial** |
| **RTs (ms)** |  |  |  |  |
| Congruent | 501.88(1.20) | 471.20(1.17) | 469.70(1.17) | 461.20(1.19) |
| Incongruent | 630.51(1.63) | 625.18(1.65) | 626.12(1.71) | 600.63(1.63) |
| Neutral | 508.33(1.19) | 481.81(1.21) | 479.51(1.17) | 469.55(1.20) |
|  |  |  |  |  |
| **Error rate** |  |  |  |  |
| Congruent | 0.032(0.0026) | 0.029 (0.0021) | 0.029 (0.0029) | 0.034 (0.0027) |
| Incongruent | 0.11(0.0074) | 0.14(0.009) | 0.15(0.010) | 0.13(0.010) |
| Neutral | 0.035(0.0026) | 0.033(0.0028) | 0.032(0.0029) | 0.036(0.0034) |

Linear mixed effects models and subsequent likelihood ratio tests assessing the main effects and the interaction of cue condition and target type on both RT and error rate were run to confirm that task dynamics of the Attention Network Task were consistent with Fan et al. (2002) and other prior research. As expected, there was a significant main effect of cue condition (χ^2^(3)=1217, *p*<.001) and of target type (χ^2^(2)=29281, *p*<.001) on RT. Additionally, there was a significant interaction between cue condition and target type on RT (χ^2^(6)=181.89, *p*<.001). The nature of this interaction is illustrated in Figure S1. We ran identical models predicting error rate which, consistent with Fan et al. (2002), revealed no main effect of cue condition (χ^2^(3)=7.75, *p*= 0.0514) but a significant main effect of target type (χ^2^(2)=407.01, *p*<.001) on error rate. The interaction between cue condition and target type was also significant (χ^2^(6)=16.91, *p*=0.00963), as illustrated in Figure S1.

We also compared pre- to post-walk reaction times and errors rates between walk conditions, averaged across all cue and target types as a measure of general processing speed and accuracy. Pre- to post-walk difference scores for both metrics can be seen in Figure S2. Linear mixed effect models revealed a significant main effect of Time on RT (χ^2^(1)=56.40, *p*<.001), such that RT decreased from pre-walk to post-walk (β =-28.45, SE=3.23, *df*=89.42, *t*=-8.80, *p*<.001; Cohen’s *d*=-0.43). There was no main effect of Condition on RT (χ^2^(1)=1.63, *p*=0.202), nor was there a significant Time by Condition interaction on RT (χ^2^(1)=0.08, *p*=0.782), meaning both groups generally responded faster after the walk with no meaningful difference in their slopes. We assume this general increase in processing speed after the walk is related to the arousal associated with two miles of low-intensity exercise (similar to our alerting results in the main manuscript). Furthermore, this provides positive evidence to suggest that the walk was not particularly fatiguing.

In terms of error rates, there were no significant main effects of Time (χ^2^(1)=0.11, *p*=0.736), Condition (χ^2^(1)=0.66, *p*=0.416), nor a Time by Condition interaction (χ^2^(1)=0.25, *p*=0.620) on error rate. Because the error rate variances were so low across time points and walk conditions (see right panel of Figure S2), error rate was excluded from any subsequent analyses. This lack of error rate variance across time and/or conditions is supported in the ANT literature (Chang et al., 2015; Kaufman et al., 2016). In fact, the seminal ANT study (Fan et al., 2002) does not include error rate calculations of each ANT index (alerting, orienting, and executive control).


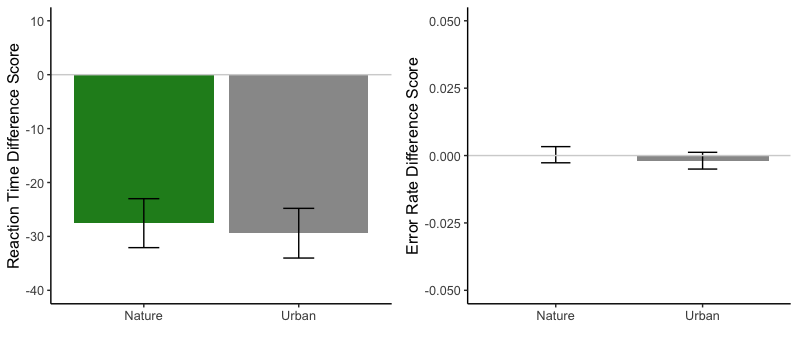
Figure S2. Reaction time (left) and error rate (right) difference scores (post-walk – pre-walk) averaged across all cue and target types as a function of walk condition. Both the nature and urban conditions showed comparable decreases in RTs from pre-walk to post-walk, and comparable (though minuscule) changes in error rate from pre-walk to post- walk. Due to the general lack of variance in error rate across the study, it was excluded from additional analyses.

Table S4. Means and standard errors of conditional and difference scores for each behavioral (reaction time) and neural (event-related potentials [ERPs]) metric generated by the ANT as a function of Walk Condition and Time.

|  | **Urban Condition** | | **Nature Condition** | |
| --- | --- | --- | --- | --- |
|  | **Pre-walk** | **Post-walk** | **Pre-walk** | **Post-walk** |
| **Overall Reaction Time (ms)** | 529.82(0.93) | 500.50(0.82) | 545.76(0.91) | 517.32(0.83) |
| **Overall Error Rate** | 0.05(0.007) | 0.05(0.005) | 0.05(0.008) | 0.05(0.006) |
|  |  |  |  |  |
| **Behavioral Metrics (Reaction Time)** |  |  |  |  |
| **Alerting** |  |  |  |  |
| No Cue (ms) | 545.47(1.79) | 525.66(1.61) | 563.40(1.77) | 542.96(1.63) |
| Double Cue (ms) | 530.30(1.92) | 496.78(1.64) | 542.68(1.85) | 511.88(1.65) |
| *Alerting RT Index (ms)* | 15.73(3.13) | 29.96(2.59) | 21.91(2.63) | 32.00(2.54) |
|  |  |  |  |  |
| **Orienting** |  |  |  |  |
| Center Cue (ms) | 529.41(1.89) | 498.32(1.64) | 543.67(1.83) | 515.05(1.66) |
| Spatial Cue (ms) | 514.04(1.83) | 481.03(1.64) | 533.18(1.78) | 499.11(1.62) |
| *Orienting RT Index (ms)* | 15.17(3.02) | 16.97(2.97) | 10.67(2.70) | 15.79(2.61) |
|  |  |  |  |  |
| **Executive Control** |  |  |  |  |
| Incongruent Target (ms) | 630.58(1.85) | 592.77(1.57) | 647.27(1.66) | 611.58(1.48) |
| Congruent Target (ms) | 482.14(1.23) | 456.24(1.11) | 496.17(1.26) | 469.30(1.12) |
| *Executive Control RT Index (ms)* | 143.24(7.48) | 133.61(6.45) | 144.92(6.83) | 138.93(4.79) |
|  |  |  |  |  |
| **Neural Metrics (ERPs)** |  |  |  |  |
| **Alerting** |  |  |  |  |
| Double Cue (µV) | 5.91(0.51) | 5.69(0.54) | 5.30(0.52) | 6.01(0.68) |
| No Cue (µV) | 5.18(0.43) | 4.70(0.55) | 4.97(0.43) | 4.68(0.57) |
| *Alerting P300 Index (µV)* | 0.73(0.28) | 0.99(0.37) | 0.34(0.29) | 1.33(0.37) |
|  |  |  |  |  |
| **Orienting** |  |  |  |  |
| Spatial Cue (µV) | 5.22(0.48) | 5.26(0.70) | 4.71(0.45) | 5.46(0.57) |
| Center Cue (µV) | 6.52(0.50) | 5.90(0.60) | 6.17(0.47) | 6.47(0.73) |
| *Orienting P300 Index (µV)* | -1.30(0.27) | -0.64(0.53) | -1.47(0.22) | -1.01(0.62) |
|  |  |  |  |  |
| **Executive Control** |  |  |  |  |
| Correct Response (µV) | 1.63(0.61) | 2.87(0.67) | 2.54(0.64) | 4.25(0.67) |
| Incorrect Response (µV) | -5.32(1.07) | -3.17(0.85) | -4.59(1.00) | -4.13(0.90) |
| *Executive Control ERN Index (µV)* | -6.95(0.91) | -6.04(0.88) | -7.13(0.97) | -8.38(0.92) |

**Exploratory Analysis**

This manuscript revealed a significant interaction between Time and Condition on amplitude of the error-related negativity (ERN), such that immersion in nature enhances the ERN while immersion in an urban environment does not. However, it is possible that there are other variables (e.g., participant age, ambient temperature, and time of day) that may, in part, be influencing these changes in ERN amplitude above and beyond environment type. While we did not have any *a priori* hypotheses regarding the impact of these variables on executive control, we report exploratory analyses to assess whether these variables meaningfully contribute to the changes in ERN amplitude that we see in the nature walk condition.

*Age*

Given the age variability of our participant cohort (Range: 18-57 years old; M= 29.43, Mdn= 26, SD= 10.52), we conducted an exploratory analysis in which we included Age as a covariate in our mixed model of interest testing the interaction between Time and Condition on ERN amplitude. Adding Age as a covariate in this model did not change the pattern of results: There was still a significant interaction between Time and Condition on ERN amplitude (χ^2^(1)=4.46, *p*=0.0348). We then ran a mixed model in which just Age (entered as a fixed effect) predicted amplitude of the ERN. There was no significant main effect of Age on amplitude of the ERN (χ^2^(1)=0.014, *p*=0.907).

*Temperature*

While there was no significant difference in average temperature between the two environmental conditions, there was variation in temperature across both conditions in the experiment (*SD*= ~12°F). Therefore, we conducted an exploratory analysis in which we included Temperature as a covariate in our mixed model of interest testing the interaction between Time and Condition on ERN amplitude. Adding temperature as a covariate in this model did not change the pattern of results: There was still a significant interaction between Time and Condition on ERN amplitude (χ^2^(1)=4.55, *p*=0.0329). We then ran a mixed model in which just Temperature (entered as a fixed effect) predicted amplitude of the ERN. There was no significant main effect of Temperature on amplitude of the ERN (χ^2^(1)=2.05, *p*=0.152).

*Time of Day*

To control for any potential differences due to time of testing, nature and urban participants were equally distributed between morning and afternoon testing sessions such that 33 participants in each group testing in the morning and 13 participants in each group tested in the afternoon. However, to ensure that our ERN results are not attributed to Time of Day, we conducted an exploratory analysis in which we included a Time of Day factor (AM versus PM) as a covariate in our mixed model of interest testing the interaction between Time and Condition on ERN amplitude. Adding Time of Day as a covariate in this model did not change the pattern of results: There was still a significant interaction between Time and Condition on ERN amplitude (χ^2^(1)=4.45, *p*=0.0348). We then ran a mixed model in which just Time of Day (entered as a fixed effect) predicted amplitude of the ERN. There was no significant main effect of Time of Day on amplitude of the ERN (χ^2^(1)=0.038, *p*=0.845).

**Re-creation of Attention Network Task Results Figure with Individual Datapoints**

While the present study was not specifically designed to test individual differences, they are an important aspect to consider in a between-subjects design involving an intervention such as a walk in a natural environment. Figure S3 is included below to allow for a visual inspection of individual differences in the Attention Network Task Results. This figure is identical to Figure 6 in the main manuscript, except that the graphs now include individual datapoints (and therefore a larger range in the *y*-axis).


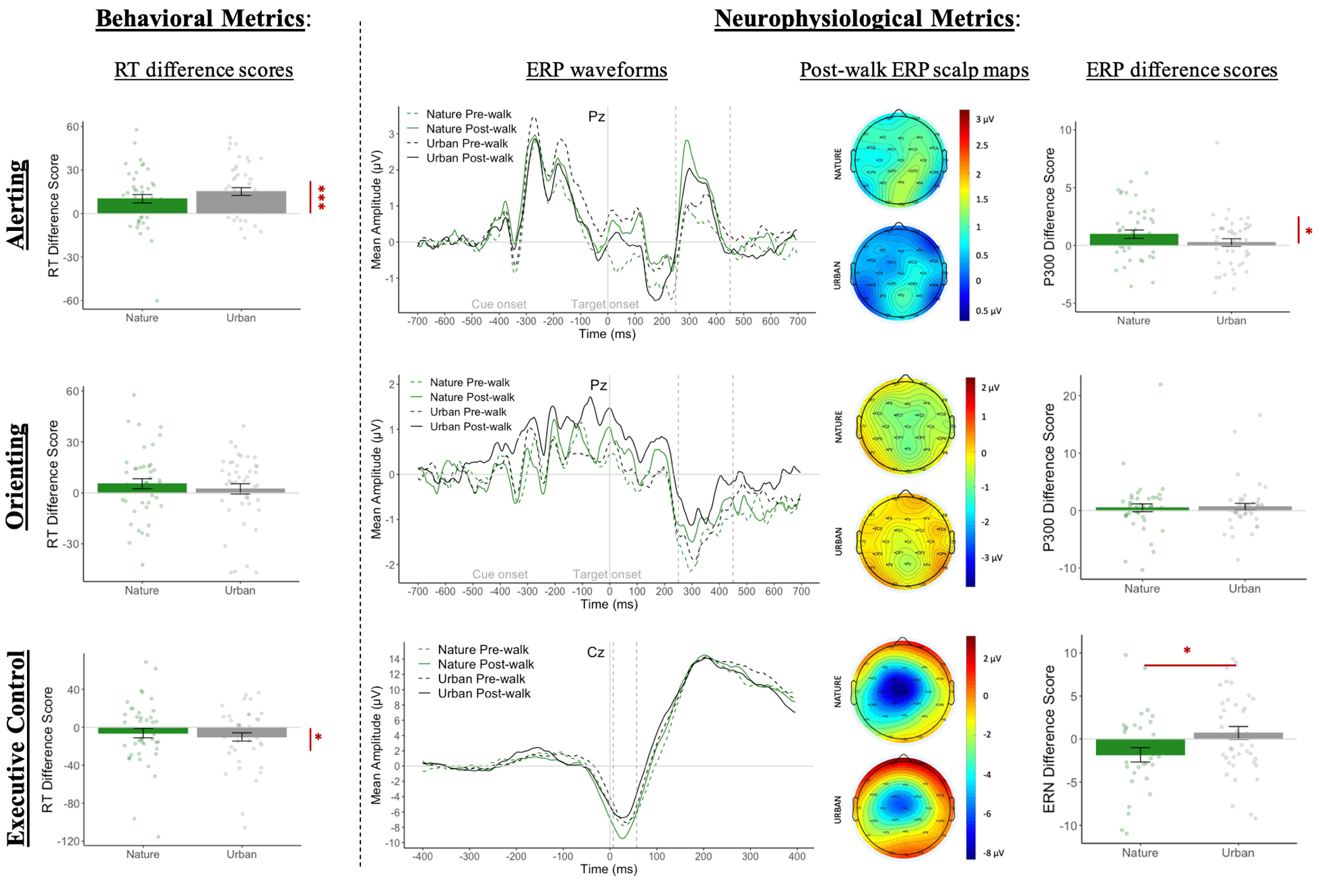


Figure S3. Attention Network Task results with individual datapoints. This figure is identical to Figure 6 in the main manuscript, except that the bar graphs now include individual datapoints. Each dot in the bar graphs represents one participant and error bars represent one standard error of the mean.

**Supplemental Materials References**

Bray, S. R., Martin Ginis, K. A., Hicks, A. L., & Woodgate, J. (2008). Effects of self‐ regulatory

strength depletion on muscular performance and EMG activation. *Psychophysiology*,

*45*(2), 337-343. <https://doi.org/10.1111/j.1469-8986.2007.00625.x>

Chang, Y. K., Pesce, C., Chiang, Y. T., Kuo, C. Y., & Fong, D. Y. (2015). Antecedent acute

cycling exercise affects attention control: an ERP study using attention network test.

*Frontiers in human neuroscience*, *9*, 1-13.

Fan, J., McCandliss, B. D., Sommer, T., Raz, A., & Posner, M. I. (2002). Testing the efficiency

and independence of attentional networks. *Journal of Cognitive Neuroscience*, *14*(3),

340-347. <https://doi.org/10.1162/089892902317361886>

Friard, O., & Gamba, M. (2016). BORIS: a free, versatile open‐source event‐logging software

for video/audio coding and live observations. *Methods in Ecology and Evolution*, *7*(11),

1325-1330.

Kaufman, D. A., Sozda, C. N., Dotson, V. M., & Perlstein, W. M. (2016). An event-related

potential investigation of the effects of age on alerting, orienting, and executive function.

*Frontiers in aging neuroscience*, *8*, 1-12.

Pasini, M., Berto, R., Brondino, M., Hall, R., & Ortner, C. (2014). How to measure the

restorative quality of environments: The PRS-11. Procedia-Social and behavioral

sciences, 159, 293-297. <https://doi.org/10.1016/j.sbspro.2014.12.375>
